# Evaluation of 2 Intervention Models to Integrate Family Planning Into Worker Health and Livelihood Programs in Egypt: A Difference-in-Differences Analysis

**DOI:** 10.9745/GHSP-D-21-00124

**Published:** 2021-12-31

**Authors:** Nahla Abdel Tawab, Elizabeth Tobey, Maryam Essam, Sara Chace Dwyer, Aparna Jain

**Affiliations:** a Population Council, Cairo, Egypt.; b Population Council, Washington, DC, USA.

## Abstract

Recent increases in fertility rates in Egypt and an increase in desired fertility among unmarried young people highlight the need for renewed attention to awareness of and demand for family planning (FP) among young people. Between 2017 and 2018, the United States Agency for International Development-funded Evidence Project tested 2 intervention models to increase awareness of and demand for FP and reproductive health (RH) services among people aged 18–35 years in Souhag and Port Said governorates, Egypt. In Souhag, FP/RH information was integrated into a 5-day livelihood training program for job seekers. In Port Said, garment factory workers received FP/RH information through trained peer educators, social and behavior change materials, and social media. Workshop participants and factory workers interested in FP services were referred to private project-trained physicians and pharmacists. We present the results of an evaluation of the impact of each intervention on young people's reported exposure to FP messages and their FP knowledge, attitudes, and behaviors. Phone interviews were conducted with participants in intervention and comparison groups before and after the intervention. In Souhag, 1,519 intervention group participants (778 at baseline; 741 at endline) and 1,082 comparison group participants (699 at baseline; 383 at endline) completed the phone interview. In Port Said 1,958 participants from intervention factories (1,145 at baseline; 813 at endline) and 1,047 participants from comparison factories (621 at baseline; 426 at endline) completed the phone interview. A difference-in-differences analysis compared intervention and comparison groups between baseline and endline. Results showed significant differences in knowledge and attitudes over time between the intervention and comparison groups in Souhag but less so in Port Said. FP use did not change among project participants in either governorate. We discuss lessons learned from integrating FP into worker health and livelihood training programs and methodological considerations for evaluating such interventions.

## Abstract (precis)

Integrating family planning and reproductive health messages into worker health programs and livelihood programs may offer a unique approach for raising young people's awareness of family planning and reproductive health.

## Teaser

**Key Findings:**

- Integrating family planning and reproductive health (FP/RH) messages into a livelihood training program for job seekers in urban Souhag, Egypt, was an effective way to improve knowledge and attitudes around FP/RH among young men and women.
- Integrating FP/RH into a worker health program for garment factory workers in Port Said, Egypt, was less effective at changing knowledge and attitudes among young male and female workers.
- In both governorates, no change in modern contraceptive use among married respondents was observed as a result of the intervention models.

**Key Implications:**

- Integrating FP/RH into worker health and livelihood programs may offer promising approaches for reaching young people in Egypt with FP/RH messages, but programmers and researchers need to consider context, reach, and sustainability when designing interventions and evaluations.

## INTRODUCTION

Egypt, a lower middle-income country, is currently facing an increase in fertility trends after several years of steady decline. Egypt's fertility rate increased from an average of 3.0 births per woman in 2008 to 3.5 births per woman in 2014, while use of any family planning (FP) method among married women of reproductive age (15–49 years) declined from 60.3% in 2008 to 58.5% in 2014.^1^ The increase in fertility was coupled with an increase in the desired number of children among never-married young people aged 15–29 years, from 2.6 children in 2009 to 2.9 children in 2014.^2^

Since the mid-1960s, Egypt has had a successful FP program due in part to strong government commitment and many years of donor support. However, several political, economic, and programmatic changes have weakened the FP program over the past decade, and as a result, 53% of married women aged 15–49 years reported no exposure to FP messages in the media in 2014, compared with 33% of married women in 2008.^1^^,^^3^ Similarly, the quality of FP counseling declined as the proportion of FP users aged 15–49 years who received information about side effects for their selected method dropped from 56% in 2008 to 48% in 2014.^1^^,^^4^ Since 2014, there has been renewed interest in FP and strong support of the national FP program from Egyptian political leadership and various government bodies.^5^^,^^6^

In 2017, 60% of Egypt's population was younger than 30 years and 24% was between age 18 and 29 years.^7^ This age group formed nearly 40% of Egypt's labor force. Reaching young men and women with correct and accurate FP information is paramount, given that they will determine the trajectory of future fertility rates in Egypt. However, Egypt's FP program has traditionally focused on married women who already have children because it is assumed that young married couples would want to have children immediately after marriage. The lack of accurate FP and reproductive health (RH) information given to young men and women makes them vulnerable to misinformation and the risk of unintended pregnancy.^8^ Many young men and women without children have misconceptions and concerns about FP side effects, particularly regarding future fertility.^3^ Moreover, sustainability of the national FP program remains a challenge, as 53% of FP users in Egypt rely on the public sector for FP service delivery.^1^ Thus, international donors have been recently supporting interventions to encourage young women (and men) to use private sector services and hence relieve some of the burden on government facilities.

Reaching young men and women with correct and accurate FP information is paramount, given that they will determine the trajectory of future fertility rates.

### Integrating FP/RH Into Worker Health and Livelihood Programs

In recent years, international companies have been collaborating with governments and nongovernmental organizations (NGOs) to implement programs to improve the health and well-being of garment factory workers.^9^^–^^12^ These programs have typically focused on occupational health and safety, but more recently, international companies have been collaborating with governments and NGOs to implement worker health programs that focus on other health topics unrelated to their occupation. Although few worker health programs have been evaluated and documented, results to date are promising. In South Africa, for example, garment workers who participated in a 6-week wellness program that included weekly aerobic exercise classes showed significant improvements in perceived quality of life and, for some, exercise-related behaviors.^9^ Gap's PACE program, which was implemented in more than 60 factories in Bangladesh, Cambodia, China, India, and Vietnam, involved a 9-module course that covered a range of topics from communication skills and decision making to general health and reproductive health.^10^ By the end of the course, improvements in women's reports of self-esteem, self-efficacy, belief in their ability to produce quality work, and their perception that their actions positively influence their workplace were observed.

Although few worker health programs have been evaluated and documented, results to date are promising.

Fewer programs have focused on improving factory workers' access to RH information and services. An evaluation of the Partnering to Save Lives project in Cambodia, for instance, found significant improvements on 7 of 14 key RH knowledge, attitude, and behavior indicators.^11^ The evaluation also found that while more women of reproductive age were using a modern contraceptive method at endline compared with baseline, women's RH knowledge was mixed. Chat!, the project's behavior change communication package that included interactive trainings, video dramas, guided discussions, and male engagement sessions was found to be positively associated with women's empowerment to discuss and use contraceptives as well as increased knowledge and use of contraception. Another program in Cambodia, the WorkerHealth intervention, combined service delivery and advocacy to improve FP/RH outcomes of female factory workers and showed a high uptake of FP/RH services.^12^

Less evidence exists on programs that aim to integrate FP/RH information and services into livelihood programs. A literature review focused on the integration of FP with microfinance and livelihood programs found some evidence suggesting integrated economic and FP programs increased knowledge and use of FP, but scant information on best practices for designing integrated FP and livelihood programs.^13^ An assessment of linked RH and livelihood programs for youth found minimal effectiveness for FP/RH outcomes and that programs often faced many challenges in providing both health and livelihood services.^14^

Between 2017 and 2018, the Population Council/Evidence Project tested 2 models to increase awareness of and demand for private FP services among young people in Souhag and Port Said governorates, Egypt. This article presents the results of an evaluation that was conducted with intervention and comparison groups, before and after the intervention. Using a difference-in-differences (DiD) analysis, the evaluation examined the effect of each intervention on young people's exposure to FP messages and their FP knowledge, attitudes, and behaviors. The article also discusses lessons learned from the implementation and research as well as methodological considerations for measuring the impact of such interventions.

## PROGRAM DESCRIPTION

### Souhag Project Setting

Souhag is a predominantly rural governorate located in the southern part of the country and has a population of nearly 4.9 million people.^7^ It is one of the poorest governorates in Egypt and has one of the highest unemployment rates in the country (14.4%).^7^ Contraceptive use is low, as 29% of married women aged 15–49 years used a modern method in 2014, while unmet need for FP is high, at 25.9%.^1^

### Souhag Intervention Overview

The intervention focused on urban male and female (married or unmarried) job seekers aged 18–35 years who had completed at least primary education, residing in 6 Souhag districts. Even though some of those job seekers may not have been married or had children, we believed it was important to raise their FP awareness at this stage before they decide to have children.

An announcement for a 5-day integrated FP/RH and livelihood training workshop (2.5 days for FP/RH and 2.5 days for livelihood skills) was shared with young people by the Women's Association for Health Improvement staff, and beneficiaries' networks. Interested young people completed an application, and those who met the age, education, and district criteria were notified of the workshop start date, which was approximately a month later.

Participants received a 5-day training in integrated FP/RH and livelihood. This training included basic FP/RH information as well as skills to assist young people in finding employment (e.g., writing a CV, presenting for a job interview). Training was offered by trained peer educators, young men and women with good communication and leadership skills. PEs offered 2 sessions simultaneously—1 for young men and 1 for young women. Each session included 25–30 participants. Social and behavior change materials (including posters, booklets, and fliers) and information on the Ma3looma social media Facebook page, which offers information and provides answers to questions about FP, were shared with participants. A total of 2,664 participants (884 young men and 1,780 young women) participated in the integrated FP/RH livelihood workshop. During the workshops, participants were given a list of names and contact information of private-sector physicians and pharmacists who had been trained by the project in FP service provision. Participants were told that they could seek additional information and services from those providers.

Participants received a 5-day training in integrated FP/RH and livelihood that included basic FP/RH information as well as skills to assist young people in finding employment.

A total of 70 private physicians and 168 pharmacists were trained in Souhag between November 2017 and March 2018. Training of physicians was for 5 days and covered FP counseling, intrauterine device (IUD) insertion, and infection control procedures. The physicians belonged to for-profit and not-for-profit (i.e., NGO) sectors. Pharmacists participated in a 1-day training on basic information about FP methods (oral pills, IUDs, injectables, and condoms) and communication skills. Regular monitoring visits to the above providers measured the quality of service provision, number of referrals, and utilization of FP services.

### Port Said Project Setting

Port Said is a coastal governate that lies in the northeast of Egypt and has a population of nearly 750,000 people.^7^ In 2014, 57% of married women aged 15–49 years in Port Said used a modern FP method.^1^ The governorate has a Free Investment Zone that includes multiple factories that produce merchandise exempt from taxes and for export. This zone includes 27 factories with nearly 38,000 employees, 60% of whom are female. Of those employed in the factories, 65% are between the ages of 18 and 35. One intervention factory has 11 subfactories that employ nearly 13,000 individuals. The remaining factories employ an average of 1,000 individuals. More than half of the factory workers employed in Port Said's Free Investment Zone reside in neighboring governorates, where FP/RH information and services are less accessible than in Port Said.

Five intervention and 2 comparison factories were selected for participation in this project. The main selection criteria were that the factory had a minimum of 1,000 workers and at least half of its labor force was female. Eight of the factories in the Free Investment Zone met the above criteria. Factories that were ready to start immediately were assigned to the intervention group while 2 of the other 3 were assigned to the comparison group. Owners of the third factory were initially reluctant to participate in the project and hence that factory was not included in the study. Eventually, that factory, along with the other 2 comparison factories, received the intervention at a later stage (not included in this study).

### Intervention Overview

The intervention, implemented in partnership with Port Fouad Childhood and Motherhood Association, was aimed at male and female workers aged 18–35 years in 5 garment factories in the Free Investment Zone of Port Said. Factory managers requested that we also raise awareness of nutrition, physical exercise, and cancer and named the project “Youth Health Project.” In collaboration with the factory management team and factory coordinators, we selected 10 to 30 male and female employees from each factory to serve as PEs and provide health messages, including FP/RH messages, to factory workers. A total of 186 PEs (107 male and 79 female) received 2.5 days of training in FP/RH and other health information, communication, and advocacy skills, and use of social media. Each trained PE was assigned an average of 40–50 factory workers with whom to share 12 FP/RH messages through face-to-face communication over 12 months. PEs shared FP/RH information with their fellow workers during lunch and tea breaks and/or bus rides and distributed social and behavior change communication materials containing FP/RH information. In larger factories, each PE was assigned to deliver messages to up to 100 workers. For sustainability purposes and to avoid work disruption, we chose to have PEs deliver messages to their coworkers on an ad hoc one-on-one basis, as opposed to a lecture format. PEs also introduced their fellow workers to Ma3looma social media Facebook page. PEs reached approximately 21,400 factory workers over a period of 1 year through face-to-face messages, social and behavior change communication materials, and Ma3looma social media Facebook page.

Peer educators were trained to provide health messages, including FP/RH messages, to their fellow factory workers.

One nurse in each factory was trained in FP counseling. PEs referred factory workers to the trained infirmary nurse for additional FP/RH information and counseling. Factory workers who expressed interest to the nurse in receiving an FP method were referred to designated private-sector physicians and pharmacists who had also been trained by the project. We trained a total of 39 physicians and 183 pharmacists in Port Said between November 2017 and March 2018. The above physicians belonged to for-profit and not-for-profit (i.e., NGO) sectors. Regular monitoring visits to those private sector providers assessed the quality of service offered, number of referrals, and utilization of FP services. Factory nurses were also asked to keep a record of the number of workers they referred to private providers each month. Several private doctors offered a discounted rate to project participants to try to attract them to their facilities.

## METHODS

### Study Design

A quasi-experimental evaluation assessed the effect of the 2 intervention models on FP knowledge, attitudes, and behaviors (Supplement). In each governorate, phone interviews were conducted with participants in the intervention and comparison groups before and after the intervention.

### Souhag Study Samples

In Souhag, the intervention group included young people who attended the 5-day integrated livelihood and FP/RH training. The Women's Association for Health Improvement obtained participants' phone numbers during workshop registration, which occurred 30–40 days before the workshop began, and asked participants if they would be willing to share their phone numbers with the research team. A total of 2,521 young men and women who registered for the training agreed to have their numbers shared with the research team. Data collectors were given a list of phone numbers of the above participants and were asked to interview 1,600 of them (800 before the workshop took place [i.e., baseline], and 800 at 3–6 months later [i.e., endline]). This target sample size was powered to assess a 5% increase in the current use of modern FP methods. A total of 1,519 phone interviews were conducted with intervention group participants (778 at baseline and 741 at endline).

The comparison group consisted of young men and women of the same age who did not participate in the intervention. These respondents were identified by random digit dialing in urban Souhag. Data collectors asked the person who answered the phone about their age and educational attainment. Those who were 18–35 and who had at least completed primary schooling were invited to participate in the study. A total of 1,082 interviews were conducted with comparison group participants (699 at baseline and 383 at endline). It is possible that the endline sample in both intervention and comparison groups may have included participants who participated in both the baseline and endline interviews for their respective treatment groups. However, those cases are unlikely to be very many and hence the data are treated as cross-sectional.

### Port Said Study Samples

In Port Said, the intervention group consisted of factory workers employed in the factories where the Youth Health Project was conducted. Before implementation of the intervention, PEs obtained verbal consent from factory workers for their telephone numbers to be shared with the research team. Facility managers then provided the research team with a database of all factory workers' phone numbers. A total of 2,946 phone numbers were shared with the research team at baseline. A total of 1,958 interviews were conducted with workers from the 5 intervention factories (1,145 at baseline and 813 at endline). This sample size was powered to assess a 5% increase in the current use of modern FP methods over the course of the intervention.

The comparison group consisted of factory workers employed in 2 factories in the Investment Zone where the intervention was not conducted. Like the intervention group, managers from those comparison factories provided the research team a database of employees' phone numbers. As the number of phone numbers provided from comparison factories was fairly small, data collectors tried all numbers that were given to them. Data collectors asked the person who answered the phone about their age and the factory where they worked. Those who were 18–35 and who were literate were invited to participate in the study. A total of 1,047 interviews were conducted with factory workers from the 2 comparison factories (621 at baseline and 426 at endline). As with the Souhag sample, we did not expect respondents in the intervention and comparison groups who participated in the baseline to have participated in their respective group's endline and vice versa, and hence the data are treated cross-sectionally.

A risk of contamination bias existed in Port Said because workers may be peers with people working at different factories and often move between factories themselves. However, it should be mentioned that locations where the intervention occurred, including bus rides and lunch breaks, were specific to workers in a given factory. Thus, workers in a comparison factory could not have received the intervention directly at the facility but could have neighbors or friends in the intervention factories who shared the messages.

### Data

Baseline interviews were conducted in Souhag from October 2017 to March 2018 and endline interviews occurred between March 2018 and September 2018. In Port Said, baseline interviews were conducted between September 2017 and September 2018 and endline interviews occurred between August 2018 and March 2019. Endline data for both the intervention and comparison group were collected in Souhag 3–6 months after each cohort completed their 5-day training course and hence endline data collection took 6 months, as the courses were held sequentially. In Port Said, endline data collection for both the intervention and comparison factories took place while the intervention was being implemented with factory workers in other factories, which occurred at staggered timelines across factories. It is noteworthy that because the intervention was meant to be sustainable and conversations between PEs and factory workers took place on an as-needed basis, there was no cutoff point for the intervention. Thus, some workers may have received their FP/RH messages one month before the endline, others may have received them 2 months earlier depending on their needs. Baseline data collection lasted 12 months and endline data collection lasted 6 months.

The same questionnaires were used in Souhag and Port Said at baseline and endline. A variety of information was gathered including respondent characteristics, use of social media, knowledge and attitudes of FP and fertility, willingness to use FP methods, and exposure to various aspects of the intervention. Experiences using FP methods were only asked of married respondents. All interviews were conducted in Arabic.

### Ethics

Ethical approval was obtained from the Population Council Institutional Review Board, and government approval was obtained to conduct the overall project. Informed consent was obtained from all respondents before each interview.

### Challenges in Data Collection

The factory worker telephone databases provided by factory managers in Port Said sometimes contained nonworking phone numbers because factory workers change their SIM cards frequently. Wrong numbers accounted for 35% of all dialed numbers in Port Said, while in Souhag they accounted for 22% of all dialed numbers. This may have led to selection bias if workers whose phone numbers were missing differed systematically in some way from those who were interviewed.

Because the survey used phone interviews (as opposed to face-to-face), the questionnaires were kept very narrow in focus. As a consequence, not all variables that may be related to the outcome variables were included in the questionnaires, which may have led to specification bias.

### Measures

The outcome variables assessed in this analysis were exposure to FP messages and FP knowledge, attitudes, and behaviors. Those outcome variables were determined based on the content of messages that were shared with workshop participants (in Souhag) or given to PEs to share with their coworkers (in Port Said). The knowledge and attitude indicators were chosen to assess the effect of the intervention in combating myths and misconceptions about FP methods, which are common among young people in Egypt,^3^ and the behavioral outcome was selected to assess whether the interventions affected contraceptive use. There were 2 exposure indicators: (1) hearing, reading, or seeing anything about FP in the preceding 6 months, and (2) ever hearing about the Ma3looma website.

#### Knowledge Outcomes

Three knowledge indicators were assessed including (1) having ever heard of an FP method; (2) knowing 3 or more modern FP methods; and (3) knowing which FP method prevents the transmission of sexually transmitted infections (STIs). Knowledge of 3 or more modern FP methods was captured by asking respondents: “Can you name FP methods that you know?” Respondents spontaneously named FP methods and were probed with “What else?” to elicit additional responses. Those who could name at least 3 methods—including pills, IUD, injectable, implant, vaginal methods (diaphragm, foam, or jelly), male condom, female condom, female sterilization, male sterilization, or emergency contraception—were categorized as knowing at least 3 modern methods. Knowledge of which FP methods prevent transmission of STIs was determined if respondents spontaneously named condoms as the method that protects against both pregnancy and STIs.

#### Attitudinal Outcomes

In terms of attitudinal questions, respondents were asked whether they agreed with the following statement: “FP methods can affect female future fertility and it may reflect negatively on future pregnancies.” Responses were captured on a Likert scale ranging from strongly agree, agree, neutral, disagree, strongly disagree, or don't know. Responses were combined into a new dichotomized variable of disagree or strongly disagree categorized as disagree versus all other responses. In the second attitudinal indicator, respondents who were not married and married respondents who reported that they or their spouses were not using an FP method at the time of the interview were asked whether they would be willing to use an FP method in the future. Those who responded “yes” were coded as willing to use an FP method in the future, while those who responded “no” or “don't know” were coded as not willing.

#### Behavioral Outcome

The behavioral indicator was whether currently married respondents (or their spouse) were using any FP method at the time of the interview.

Other variables used to adjust the analysis included respondents' background characteristics, namely age, residence, sex, education, employment status, marital status, and number of living children.

### Analysis

For each governorate, descriptive statistics were calculated for the intervention and comparison groups at baseline. Chi-squared tests were used to assess differences in characteristics at baseline between the comparison group and the intervention group. To assess the effect of the intervention on knowledge, attitudes, and behavior in each governorate, a DiD analysis was conducted. The DiD analysis used for this study can be represented by the following equation:

$$\delta_{DD}=\left({\bar{Y}}_{Endline}^{Intervention}-{\bar{Y}}_{Endline}^{Intervention}\right)-({\bar{Y}}_{Endline}^{Comparison}-{\bar{Y}}_{Baseline}^{Comparison})$$

To assess the effect of the intervention on knowledge, attitudes, and behavior in each governorate, a DiD analysis was conducted.

The DiD estimate $\delta_{DD}$measures the change in outcomes that can be attributed to the intervention, and $\bar{Y}$ represents the proportion of each sample with the desired outcome for a given variable.

By subtracting the change over time in the outcome variable for the comparison group from the change over time in the intervention group, the DiD accounts for any change that might have occurred in the population naturally and thus cannot be attributed to the intervention. The DiD requires an assumption that this trend would be similar in the intervention and comparison groups. Unadjusted and adjusted DiD estimates were calculated to account for differences in respondent characteristics between groups.

## RESULTS

Table 1 presents the background characteristics of respondents in the intervention and comparison groups in Souhag and Port Said at baseline. In Souhag, respondent background characteristics were significantly different between the comparison and intervention groups for age, sex, education completed, employment status, marital status, and number of children at baseline. In Port Said, similar significant differences were observed for age, education completed, marital status, number of children, and having ever heard of an FP method between the comparison and intervention groups.

**TABLE 1.** Background Characteristics of Respondents in a Study to Assess the Effect of 2 Intervention Models on Family Planning Knowledge, Attitudes, and Behaviors in Souhag and Port Said, Egypt

	Souhag, %		Port Said, %	
	Intervention (n=778)	Comparison (n=699)	Intervention (n=1,145)	Comparison (n=621)
Age,^a,b^ years				
18–19	12.2	6.4	10.5	9.8
20–24	44.7	25.9	25.9	36.1
25–29	27.9	30.2	33.5	31.7
30–35	15.2	37.3	30.1	22.2
Residence				
Urban	67.1	62.5	83.2	84.5
Rural	32.9	37.5	16.8	15.5
Sex^a^				
Male	35.6	47.4	77.4	78.7
Female	64.4	52.6	22.6	21.3
Education completed^a,b^				
Never attended/less than primary	0.4	0.1	4.2	2.1
Primary/preparatory	5.5	0.7	10.7	7.4
Secondary/intermediary	62.1	58.7	68.9	67.3
University/higher	32.0	40.5	16.2	23.2
Employment status^a^				
Currently employed	30.7	43.8	100	100
Unemployed and searching	36.8	24.5	0	0
Out of labor force	32.5	31.8	0	0
Marital status^a,b^				
Never married	55.5	34.9	40.5	48.0
Engaged/marriage contract	8.2	6.9	14.4	17.4
Married	33.9	56.4	42.8	32.1
Widowed/divorced/ separated	2.3	1.9	2.3	2.6
Number of living children^a,b^				
0	71.3	50.6	63.4	73.8
1	10.2	11.0	15.6	13.9
2	8.9	17.0	15.9	9.0
3 or more	9.6	21.3	5.1	3.4
Ever heard of a family planning method^b^				
Yes	83.3	83.1	76.5	68.3
No/don't know	16.7	16.9	23.5	31.7

a Statistically significant for Souhag at *P*<.01.

b Statistically significant for Port Said at *P*<.01.

Table 2 presents DiD estimates for all outcome indicators in Souhag, including adjusted estimates to account for differences between groups. Results of the DiD analysis show significant differences in changes over time between the intervention and comparison groups on 7 of the 8 indicators, with the intervention group showing improvements across all 7 indicators. For example, the DiD estimate for having heard, read, or seen anything about FP in the 6 months preceding the survey was 41 percentage points and 28 percentage points for having heard of the Ma3looma website. In terms of knowledge indicators, the DiD estimates were 13 percentage points for having ever heard of an FP method, 22 percentage points for knowing 3 or more modern FP methods, and 36 percentage points for knowing that condoms are the FP method that also prevents against STIs. For attitudinal indicators, the DiD was 19 percentage points for disagreeing with “FP methods can affect female fertility and it may reflect negatively on future pregnancies.” The DiD for being willing to use FP in the future showed that among those not currently using an FP method, there was a slight, but significant increase for the intervention group compared with the comparison over time, at 7 percentage points. However, regarding whether the intervention affected behavior, no significant difference was found in the change in the proportion of married respondents (or their spouses) who were currently using FP over time compared with change observed in contraceptive use among respondents in comparison factories.

**TABLE 2.** DiD Estimates of Young People's Exposure to FP and Their FP Knowledge, Attitudes, and Behaviors in Souhag, Egypt

	Percentage^a^		DiD Results	
Group	Baseline	Endline	Percentage Point Estimate	Adjusted Percentage Point Estimate^b^
Exposure outcomes				
Heard, read, or seen anything about FP in the last 6 months				
Intervention	28.1	74.1	41.3^c^	40.2^c^
Comparison	18.9	23.5		
Has heard of Ma3looma website				
Intervention	17.6	49.4	28.0^c^	27.7^c^
Comparison	8.7	12.5		
Knowledge outcomes				
Ever heard of an FP method				
Intervention	83.3	96.1	12.6^c^	11.5^c^
Comparison	83.1	83.3		
Knows 3 modern FP methods				
Intervention	56.0	83.0	22.0^c^	19.6^c^
Comparison	49.6	54.6		
Knows FP method that prevents sexually transmitted infections				
Intervention	18.0	56.8	36.4^c^	35.0^c^
Comparison	13.0	15.4		
Attitudinal outcomes				
Disagrees with “FP methods can affect female fertility and it may reflect negatively on future pregnancies”				
Intervention	52.6	73.5	18.6^c^	17.8^c^
Comparison	46.2	48.6		
Willing to use FP in the future (among individuals not currently using an FP method)^d^				
Intervention	88.8	94.5	6.5^e^	6.0^e^
Comparison	85.9	85.2		
Behavioral outcomes (among married individuals who are not currently pregnant/whose wives are not currently pregnant)				
Currently using FP^f^				
Intervention	62.6	62.9	0.1	−3.0
Comparison	62.4	62.5		

Abbreviations: DiD, Difference-in-Differences; FP, family planning.

a Sample sizes at baseline were n=778 for the intervention group and n=699 for the comparison group. Sample sizes at endline were n=741 for the intervention group and n=383 for the comparison group.

b Adjusted for age, residence, gender, education, employment, marital status, and number of children.

c *P*≤.01.

d Sample sizes at baseline were n=644 for the intervention group and n=490 for the comparison group. Sample sizes at endline were n=587 for the intervention group and n=263 for the comparison group.

e *P*≤.05.

f Sample sizes at baseline were n=214 for the intervention group and n=335 for the comparison group. Sample sizes at endline were n=245 for the intervention group and n=192 for the comparison group.

Results of the DiD analysis show significant differences in changes over time between the intervention and comparison groups on 7 of the 8 indicators in Souhag.

### Port Said

Table 3 presents DiD estimations in Port Said for all outcome indicators. Few significant differences were found in intervention exposure, knowledge, attitudes, and behavior outcomes for changes observed over time in the intervention group compared with those in the comparison group. The DiD estimate for the proportion of respondents who disagreed with the statement “FP methods can affect female fertility and it may reflect negatively on future pregnancies” was significant at 8.3 percentage points. Although the proportion of the intervention group disagreeing with the statement only increased by 2.7 percentage points, the proportion of the comparison group disagreeing dropped by 5.6 percentage points, leading to a significant DiD estimate of 8.3 percentage points. Similar to Souhag, there was no significant difference in the behavioral outcome of current use of FP among married respondents, with a DiD estimate of −1.7 percentage points.

**TABLE 3.** DiD Estimates of Factory Workers' Exposure to FP and Their FP Knowledge, Attitudes, and Behaviors in Port Said, Egypt

	Percentage^a^		DiD Results	
Group	Baseline	Endline	Percentage Point Estimate	Adjusted Percentage Point Estimate^b^
Exposure outcomes				
Heard, read, or seen anything about FP in the last 6 months				
Intervention	16.2	17.2	−4.9	−4.7
Comparison	16.9	22.8		
Has heard of Ma3looma website				
Intervention	6.1	10.2	−3.8	−4.0
Comparison	6.0	13.8		
Knowledge outcomes				
Ever heard of an FP method				
Intervention	76.5	74.3	−6.5	−7.8^c^
Comparison	68.3	72.5		
Knows 3 modern FP methods				
Intervention	34.5	38.9	0.8	−0.3
Comparison	27.7	31.2		
Knows FP method that prevents sexually transmitted infections				
Intervention	12.9	15.1	−0.1	−0.9
Comparison	14.3	16.7		
Attitudinal outcomes				
Disagrees with: “FP methods can affect female fertility and may reflect negatively on future pregnancies”				
Intervention	41.0	43.7	8.3^c^	9.2^c^
Comparison	43.6	38.0		
Willing to use FP in the future (among individuals not currently using an FP method)^d^				
Intervention	66.9	71.6	2.1	2.0
Comparison	74.3	77.0		
Behavioral outcomes (among married individuals who are not currently pregnant/whose wives are not currently pregnant)				
Currently using FP^e^				
Intervention	62.6	69.9	−1.7	−0.9
Comparison	59.5	68.6		

Abbreviations: DiD, Difference-in-Differences; FP, family planning.

a Sample sizes at baseline were n=1,145 for the intervention group and n=621 for the comparison group. Sample sizes at endline were n=813 for the intervention group and n=426 for the comparison group.

b Adjusted for age, residence, gender, education, employment, marital status, and number of children.

c *P*≤.05.

d Sample sizes at baseline were n=896 for the intervention group and n=521 for the comparison group. Sample sizes at endline were n=564 for the intervention group and n=343 for the comparison group.

e Sample sizes at baseline were n=398 for the intervention group and n=168 for the comparison group. Sample sizes at endline were n=356 for the intervention group and n=121 for the comparison group.

## DISCUSSION

The purpose of this study was to assess the effect of 2 intervention models on changes in FP knowledge, attitudes, and behaviors among young job seekers in urban Souhag and young factory workers in Port Said, Egypt. Results from the DiD analysis suggest that the intervention in Souhag was effective in improving FP/RH indicators related to exposure, knowledge, and attitudes. However, in Port Said, there was only 1 significant DiD estimate for the statement “FP methods can affect female fertility and it may reflect negatively on future pregnancies.” The findings from this implementation research also present valuable lessons for future FP/RH worker health and livelihood training interventions and evaluations.

### Implementation Successes and Challenges

While the evidence base on programs that aim to integrate FP/RH information and services into livelihood programs is small, results from our DiD analysis suggest that the integrated livelihood and FP intervention was effective in increasing knowledge and positive attitudes toward FP among young people in urban Souhag. The results from this analysis are similar to those from a literature review on the integration of FP into microfinance and livelihood programs that found some evidence suggesting increased knowledge and use of FP after these programs.^13^

Results suggests that the integrated livelihood and FP intervention was effective in increasing knowledge and positive attitudes toward FP among young people in urban Souhag.

Part of the success in Souhag may be due to the highly structured nature of the intervention. For example, PEs and workshop participants met on predetermined dates, messages were delivered according to a largely fixed schedule, and all participants completed the 5 days of training and received the same FP messages during the 5-day workshop (2.5 days for livelihood skills and 2.5 days for FP/RH information). However, similar to challenges faced in the implementation of the workplace health program HERproject in Bangladesh, high turnover of PEs can be a challenge.^16^ The Souhag livelihood training program faced similar challenges, with PEs opting for more stable, full-time jobs rather than the part-time, temporary PE positions. Of 60 PEs who had been trained by the project in Souhag, 18 left the project by the end of the first year. The results, coupled with the implementation learnings, suggest that highly structured programs may be more effective in improving RH/FP knowledge and attitudes, but additional research is needed to identify best practices for retaining PEs or targeting permanent employees as PEs.

Previous evaluations of worker health programs that offered health education to factory workers through PEs have been shown to raise awareness of general and reproductive health, use of clinic services, and workers' hygiene and other health behaviors improved.^15^^–^^17^ Our DiD analysis, however, found that the worker health intervention implemented in Port Said was not effective in changing factory workers' knowledge, attitudes, or behaviors. Several programmatic issues during the implementation of the intervention in Port Said may have minimized the worker health program's effect. The intervention was implemented with PEs who worked in various capacities in the factories, and this may have reduced the time available to convey FP/RH messages to their coworkers. The time available for PEs to communicate messages was limited to tea and lunch breaks or bus rides. As a result, very few PEs were able to speak to the target number of colleagues to reach in a given month. In larger factories, the PE to worker ratio was 1:100, which made it even more difficult for PEs to reach their target workers each month. Not surprisingly, due to these challenges and because the intervention/comparison surveys were conducted at the facility rather than the individual level, the proportion of factory workers in intervention factories who reported exposure to FP/RH messages at endline was quite small, at 17%. Moreover, messages were communicated by PEs on an ad hoc basis, depending on the needs and interests of individual workers. Thus, not all workers received the same messages in a given month.

The above challenges in integrating FP/RH messages into workers' health programs are not uncommon. In Bangladesh, the PEs in the HERhealth project disseminated messages during work breaks similar to Port Said and turnover of PE was also an implementation challenge.^16^ However, a similar DiD analysis found the HERhealth project had a positive effect on female workers' health knowledge and behaviors. We hypothesize that differences in how the 2 projects were implemented may help explain why the intervention was not effective in Port Said. First, the ratio of PE to female factory workers under the HERhealth project was between 1:21 and 1:26, whereas the ratio was up to 1:100 in Port Said. Second, the HERhealth PE training in Bangladesh was covered over 10 weeks, versus 5 days plus monthly sessions thereafter in Port Said. Additional research is needed to understand the essential program components that will lead to better results for workplace programming.

The DiD analysis did not reveal changes in FP behaviors at either intervention site, possibly due to cost. The project referred participants to private sector providers, some of whom may have had relatively high fees that project participants may not have been able to afford. In Port Said, more than half of the workers came from neighboring governorates and were reluctant to miss work hours to visit the private providers trained by the project and who were located within the Port Said governorate. The model in Port Said also involved a 2-step referral process (from PEs to factory nurses for counseling and from factory nurses to private sector providers for a method), which may have created barriers to workers' access to FP services. In response to the above challenges, the project eventually facilitated setting up a Women's Health clinic to provide FP/RH services to more than 20,000 female workers in the Investment Zone. An evaluation from workplace programming found that substantial changes in menstrual hygiene management were observed because the intervention also worked with factory clinics to expand access to sanitary menstrual hygiene products in addition to disseminating menstrual hygiene health messages via PE.^17^ Thus, the addition of the Women's Health clinic in Port Said's Investment Zone may complement the education in a similar way and increase use of FP/RH services. Implementation research on the acceptability and effectiveness of this intervention will be necessary for expanding the evidence base for including service provision in worker health education for FP/RH.

The DiD analysis did not reveal changes in FP behaviors at either intervention site, possibly due to cost.

In both governorates, more than half of married participants in this study had no children. These married participants with no children were far less likely to be using FP (3%) than their counterparts with children (76%, data not shown), which may be due in part to traditional values that favor having at least 1 child before using contraception.^3^ This may explain why significant increases in FP use were not seen, despite increases in the proportion of respondents who would be willing to use FP in the future in Souhag.

### Research Limitations

#### Souhag

In Souhag, although phone numbers were obtained directly from workshop participants during registration, participants often shared their phones with other family members so when the research team called, the participant may not have been in possession of the phone. The male/female distribution of survey participants differed only slightly from the makeup of the workshop participants (70% female to 30% male for the survey versus 60% female and 40% male for the workshop). In Souhag, the sampling strategy may have led to some biases in the study findings. Participants at baseline and endline were sampled cross-sectionally, so the same respondents were not necessarily interviewed at baseline and endline. However, some may have been interviewed at both time points, although this proportion could not be determined. Regardless, DiD estimations are calculated the same way for both panel and cross-sectional data, and quasi-experimental design using comparison and intervention groups interviewed before and after the intervention mitigates the selection bias associated with the sampling. Although the intervention participants were not selected randomly, efforts were made to ensure that the intervention and comparison groups were similar in terms of age, educational attainment, and geographic residence. However, there were significant differences between the comparison and intervention groups. For example, the comparison group was older than the intervention group, which might have been more susceptible to learning new information, attitudes, and behaviors. To mitigate the effects of any confounding variables and reduce any bias that may have been introduced due to the groups being significantly different, the DiD analyses were adjusted for respondent characteristics.

#### Port Said

In Port Said, the project experienced difficulty in determining intervention cutoff points and reaching survey respondents. As the content of messages that were communicated to factory workers varied depending on the needs of each worker, there was no clear cutoff point for the intervention. As mentioned earlier, the duration between receiving FP/RH messages and the endline interview varied for different workers depending on their needs, as the interventions and surveys were conducted on a factory rather than an individual level. In addition, worker movement across garment factories is common as workers move from one factory to the other to respond to purchase orders. Some workers may have moved from intervention to comparison factories and vice versa. In addition, workers from both intervention and comparison factories may live in similar neighborhoods and take similar buses to and from work, so diffusion of the intervention to the comparison group may have occurred. These 2 factors may have contributed to the lack of significant results between the intervention and comparison groups. Similarly, worker movement within the same factory occurs across factory halls/departments, some of which did not have a trained PE, which may have contributed to the lack of significant findings. Also, it should be noted that phone numbers of factory workers that were shared with the research team belonged to factory workers in the entire factory and not necessarily those workers who were reached by the PEs. It is also important to note that the endline sample in Port Said included an overrepresentation of workers from larger factories, which had a low PE to worker ratio. Finally, female factory workers were less likely to answer phone calls from the project's research staff, which skewed the distribution of male to female respondents. The typical sex distribution of factory workers in Port Said is 40% males and 60% females, while the distribution of respondents in this study was 78% males and 22% females.

The above challenges in conducting the research in Port Said, along with the sampling strategy, may have led to some biases in the study findings. Similar to Souhag, participants were sampled cross-sectionally before and after the intervention, so the same respondents were unlikely to be interviewed at both baseline and endline, although some may have been. Additionally, although the intervention occurred at the facility level and not the individual level, efforts were made to ensure that the factories chosen for the intervention and comparison groups were similar. The comparison and intervention groups were similar in terms of age as well. Finally, although the 2 groups were significantly different on some respondent characteristics, which may have introduced bias into the results, we adjusted the DiD analyses for individual-level characteristics to mitigate the effects of any confounding variables on the results.

## CONCLUSION

Worker health and livelihood training programs may offer a unique venue for conveying FP/RH messages to young people if the context is taken into consideration. For example, in factory settings, the number of PEs in a given factory should be proportionate to the number of workers in that factory. PEs need to be selected from workers who have more control over their time or whose work involves some mobility (e.g., line supervisors or human resources officers). In addition, future programs could consider innovative ways for PEs to stand out and be more visible and identifiable by their coworkers (e.g., by having PEs wear special buttons), especially in larger factories where the PE to worker ratio may be low, which could improve information exchange. PEs could also complement face-to-face communication with social and behavior change materials and social media platforms like Facebook and WhatsApp. However, close attention would be needed in identifying ways to protect workers' privacy and confidentiality if sensitive information is exchanged over social media platforms. Finally, raising FP/RH awareness of factory workers cannot be left to PEs alone. The factory nurse could play a more proactive role in conducting FP/RH education seminars and integrating FP/RH messages to factory workers who are seeking other services such as first aid. The role of nurses could be expanded to include overseeing health education activities provided by PEs, training PEs, and providing one-on-one FP/RH counseling to factory workers. Factory management also needs to take ownership of worker health programs and establish structures and systems within the factory to sustain the program.^18^

Likewise, livelihood programs could offer an opportunity for raising young people's awareness about FP/RH if the 2 components are integrated efficiently. The impact of one-off integrated trainings on reproductive behaviors could be enhanced by linking participants to designated FP services that are accessible to workshop participants. However, sustainability of those integrated livelihood programs (i.e., getting governments or employers to support the costs of the FP/RH component) remains a challenge. Policy makers, donors, program managers, and business owners need to recognize that linking FP/RH with livelihood programs benefits both sectors.^13^

More research is needed to demonstrate the economic benefits of integrating FP/RH into livelihood and worker health programs. Additional research on the effect of respondents' relationships, communication about FP/RH topics with partners, and gender attitudes and norms would provide a more robust examination of any changes in behavior and attitudes over time. Also, more implementation research is needed to identify key components of successful integrated FP/RH and livelihood/worker health programs, such as length of PE training and ideal ratios of PEs to workers, and to evaluate the long-term impact of those programs on workers' well-being, productivity, and retention as well as the return on investment of such programs. Additionally, research designs to evaluate the impact of worker health programs should take into consideration the mobile nature of work in factory settings, the casual nature of discussions between PEs and workers, and the frequent changing of SIM cards by factory workers. Mechanisms for tracking workers' mobility within and across factories and obtaining updated lists of workers' phone numbers should be developed in future research studies. Lastly, additional research is needed on the acceptability and effectiveness of adding a Women's Health clinic to the Investment Zone of Port Said, in terms of the use of FP/RH services among workers.

## Supplementary Material

GHSP-D-21-00124-Supplement.pdf

## Notes

**Peer Reviewed**

**First published online:** October 26, 2021.

## References

[1] Egypt, Ministry of Health and Population (MOHP), El-Zanaty and Associates, ICF International. Egypt Demographic and Health Survey 2014. MOHP and ICF International; 2015. Accessed August 20, 2021. https://dhsprogram.com/pubs/pdf/FR302/FR302.pdf

[2] Roushdy R Sieverding M. Summary Report: Panel Survey of Young People in Egypt (SYPE) 2014—Generating Evidence for Policy, Programs, and Research. Population Council; 2015. Accessed August 20, 2021. https://www.popcouncil.org/uploads/pdfs/2015PGY_SYPE-Summary.pdf

[3] Abdel-Tawab N Attia S Bader N Roushdy R El-Nakib S Oraby D. Fertility Preferences and Behaviors Among Younger Cohorts in Egypt: Trends, Correlates, and Prospects for Change. Population Council, The Evidence Project; 2020. Accessed August 20, 2021. https://evidenceproject.popcouncil.org/resource/egypt-young-fertility-preferences-behaviors/

[4] El-Zanaty F Way A. Egypt Demographic and Health Survey 2008. Ministry of Health, El-Zanaty and Associates, and Macro International; 2009. Accessed August 20, 2021. https://dhsprogram.com/pubs/pdf/FR220/FR220.pdf

[5] State Information Service. Constitution of The Arab Republic of Egypt. Government of Egypt; 2014. Accessed August 20, 2021. https://www.sis.gov.eg/Newvr/Dustor-en001.pdf

[6] Egypt. Ministry of Health and Population. National Population Strategy 2015–2030. Report in Arabic. National Population Council; 2014.

[7] Central Agency for Public Mobilization and Statistics (CAPMAS). Egypt Census 2017. In Arabic. CAPMAS; 2017. Accessed August 20, 2021. http://www.capmas.gov.eg/Pages/ShowPDF.aspx?page_id=/Admin/Pages%20Files/201710914947book.pdf

[8] Roudi-Fahimi F El Feki S. Facts of Life: Youth Sexuality and Reproductive Health in the Middle East and North Africa. Population Reference Bureau; 2011. Accessed August 20, 2021. https://www.prb.org/wp-content/uploads/2011/07/facts-of-life-youth-in-middle-east.pdf

[9] Edries N Jelsma J Maart S. The impact of an employee wellness programme in clothing/textile manufacturing companies: a randomised controlled trial. BMC Public Health. 2013;13(1):25. 10.1186/1471-2458-13-25. 23311458 PMC3574831

[10] Nanda P Mishra A Walia S Weiss E Abrahamson J. Advancing Women, Changing Lives: An Evaluation of Gaps Inc.'s P.A.C.E. Program. International Center for Research on Women; 2013. Accessed August 20, 2021. https://www.icrw.org/wp-content/uploads/2016/10/PACE_Report_PRINT_singles_lo.pdf

[11] Nicewinter JP Ramage I. Endline Survey of Reproductive, Maternal, and Neonatal Health Knowledge, Attitudes and Practices Among Garment Factory Workers. Partnering to Save Lives and Angkor Research; 2018. Accessed August 20, 2021. https://www.dfat.gov.au/sites/default/files/cambodia-partnering-to-save-lives-endline-survey-report-june-2018.pdf

[12] Bajracharya A. Findings from the WorkerHealth post-intervention qualitative assessment. Presentation at the WorkerHealth End of Project meeting; February 22, 2018. Accessed August 20, 2021. https://knowledgecommons.popcouncil.org/departments_sbsr-rh/1379/

[13] Marcus R Bercu C Norton M Mutunga C. Improving the evidence for integrated family planning and economic growth programming: a synthesis of the evidence. Gates Open Research. 2019;3:1497. 10.12688/gatesopenres.12994.1

[14] Esim S Malhotra A Mathur S Duron G Johnson-Welch C. Making It Work: Linking Youth Reproductive Health and Livelihoods. International Center for Research on Women; 2001. Accessed August 20, 2021. https://www.icrw.org/wp-content/uploads/2016/10/Making-it-Work-Linking-Youth-Reproductive-Health-and-Livelihoods.pdf

[15] Yeager R. HERproject: Health Enables Returns: The Business Returns From Women's Health Programs. BSR and Levi Strauss Foundation; 2011. Accessed August 20, 2021. https://www.bsr.org/reports/HERproject_Health_Enables_Returns_The_Business_Returns_from_Womens_Health_Programs_081511.pdf

[16] Hossain MdI. Al Mahmud A Bajracharya A Rob U Reichenbach R. Evaluation of the Effectiveness of the HERhealth Model for Improving Sexual and Reproductive Health and Rights Knowledge and Access of Female Garment Factory Workers in Bangladesh. Population Council, The Evidence Project; 2017. Accessed August 20, 2021. https://evidenceproject.popcouncil.org/resource/herhealth-model-evaluation/

[17] Hossain MdI Chace Dwyer S Bajracharya A Jain A. Reaching women at work: improving reproductive health knowledge and outcomes for female factoryworkers throughworkplace programs in Bangladesh, results from difference and differences analyses. Presentation at the Population Association of America 2021 Annual Meeting; May 5, 2021.

[18] Rodehau C Wofford D Almeida K Goldenberg E. Strengthening Factory Health Systems Under Levi Strauss & Co.'s Worker Well-being Initiative in Egypt. Population Council, Evidence Project; 2015. Accessed August 20, 2021. https://evidenceproject.popcouncil.org/wp-content/uploads/2016/03/Levis-Brief_3.22.16.pdf

