# Supplementary material for: Evaluation of 2 Intervention Models to Integrate Family Planning Into Worker Health and Livelihood Programs in Egypt: A Difference-in-Differences Analysis
Source: Glob Health Sci Pract. 2021 Dec 31;9(4):804–17. doi: 10.9745/GHSP-D-21-00124 (PMC8691888; doi:10.9745/GHSP-D-21-00124)
Supplement: GHSP-D-21-00124-Supplement.pdf [file GHSP-D-21-00124-Supplement.pdf]

Supplement to: Abdel Tawab N, Tobey E, Essam M, Chace Dwyer S, Jain A. Evaluation of 2 intervention models to integrate family planning into worker health and livelihood programs in Egypt: a difference-in-differences analysis. *Glob Health Sci Pract*. 2021;9(4). https://doi.org/10.9745/GHSP-D-21-00-124

Supplement Table 1. Intervention characteristics in Souhag and Port Said

	Souhag	Port Said
Intervention model	Integration of FP/RH into livelihood training	Integration of FP/RH into worker health programs
Participant Selection Criteria	Urban male and female job seekers aged 18-35 who have completed at least primary education.	Male and female garment factory workers aged 18-35 who are literate.
Location of intervention	Workshops offered to participants in six districts in urban Souhag	Five garment factories in the Free Investment Zone
Recruitment procedures	Announcement for a 5-day training shared by WAHI staff and beneficiaries' networks. Interested young people who fulfilled selection criteria were notified of the workshop start date.	Trained peer educators received a list of names of factory workers whom s/he was assigned to. Those workers were 18-35 years old and were literate. The number of workers on the list ranged from 40 –100 depending on the size of factory.
Components of the intervention	Trained male and female peer educators (PEs) offered a five-day integrated FP livelihood training workshop and shared SBCC materials with participants.<br><br>Participants were given a list of names and contact information of private sector service providers who had been trained by the project in FP service provision.	Trained PEs shared messages with their fellow workers through face to face communication and SBCC materials and referred those who needed additional FP/RH information to the trained factory infirmary nurse.<br><br>Factory infirmary nurse referred those who needed a FP/RH service to trained private sector private providers.

Supplement to: Abdel Tawab N, Tobey E, Essam M, Chace Dwyer S, Jain A. Evaluation of 2 intervention models to integrate family planning into worker health and livelihood programs in Egypt: a difference-in-differences analysis. *Glob Health Sci Pract*. 2021;9(4).
https://doi.org/10.9745/GHSP-D-21-00-124

Supplement Table 2. Study design in Souhag and Port Said

	Souhag	Port Said
Design	Cross sectional	Cross sectional
Intervention group	Young men and women aged 18-35 who attended the five-day integrated livelihood and FP/RH training	Factory workers aged 18-35 employed in five intervention factories where the Youth Health Project was implemented.
Comparison group	Young men and women of the same age who did not participate in the intervention.	Factory workers employed in the factories where the Youth Health Project was not conducted.
Recruitment of research participants	WAHI obtained participants' phone numbers during workshop registration (30-40 days before workshop)	Facility managers provided the research team with a database of all factory workers' phone numbers.
Recruitment of comparison group	Random digit dialing in urban Souhag	Managers from two comparison factories provided the research team with a database of employees' phone numbers.
Sample of research participants	1,519 phone interviews (778 at baseline and 741 at endline)	1,958 phone interviews (1,145 at baseline and 813 at endline)
Sample of comparison group	1,082 phone interviews (699 at baseline and 383 at endline)	1,047 phone interviews (621 at baseline and 426 at endline)
Baseline	October 2017 to March 2018<br><br>Data collectors were given a list of phone numbers and asked to interview at baseline (before the workshop took place)	September 2017 to September 2018
Endline	March 2018 to September 2018<br><br>Data collectors were given a list of phone numbers and asked to interview at endline (3-6 months after workshop	August 2018 to March 2019
